# Gene methylation as a powerful biomarker for detection and screening of non-small cell lung cancer in blood

**DOI:** 10.18632/oncotarget.15919

**Published:** 2017-03-06

**Authors:** Bao-hua Wang, Yan-yu Li, Jin-zhu Han, Lian-ya Zhou, Ying-qian Lv, He-lin Zhang, Li Zhao

**Affiliations:** ^1^ Department of Thoracic Surgery, The Second Hospital of Heibei Medical University, Shijiazhuang 050000, China; ^2^ Department of General Surgery, The Second Hospital of Heibei Medical University, Shijiazhuang 050000, China; ^3^ The Second Department of Oncology, The Second Hospital of Heibei Medical University, Shijiazhuang 050000, China

**Keywords:** gene methylation, NSCLC, blood, biomarkers

## Abstract

DNA methylation has been reported to become a potential powerful tool for cancer detection and diagnosis. However, the possibilities for the application of blood-based gene methylation as a biomarker for non-small cell lung cancer (NSCLC) detection and screening remain unclear. Hence, we performed this meta-analysis to evaluate the value of gene methylation detected in blood samples as a noninvasive biomarker in NSCLC. A total of 28 genes were analyzed from 37 case-control studies. In the genes with more than three studies, we found that the methylation of *P16*, *RASSF1A*, *APC*, *RARβ*, *DAPK*, *CDH13*, and *MGMT* was significantly associated with risks of NSCLC. The methylation statuses of *P16*, *RASSF1A*, *APC*, *RARβ*, *DAPK*, *CDH13*, and *MGMT* were not linked to age, gender, smoking behavior, and tumor stage and histology in NSCLC. Therefore, the use of the methylation status of *P16*, *RASSF1A*, *APC*, *RARβ*, *DAPK*, *CDH13*, and *MGMT* could become a promising and powerful biomarker for the detection and screening of NSCLC in blood in clinical settings. Further large-scale studies with large sample sizes are necessary to confirm our findings in the future.

## INTRODUCTION

Non-small cell lung cancer (NSCLC) accounts for approximately 80% of lung cancer which has become the top cause of cancer deaths in the world [1]. NSCLC includes adenocarcinoma (AC), squamous cell carcinoma (SCC), large cell carcinoma, and adenosquamous carcinoma [2, 3]. NSCLC is commonly diagnosed by a comprehensive evaluation of symptoms, medical imaging, assessment of the levels of serum tumor biomarkers, and, eventually, cytological examination. Imaging methods, such as chest X-rays and computed tomography (CT), are widely used, but they do not have sufficient sensitivity and specificity to detect the early stages of NSCLC [4]. Reportedly, PET imaging has a better potential for detection of NSCLC but is characterized by the high costs of analysis [5, 6]. Many studies suggest that the combined detection of several tumor markers, including CEA, PRO-GRP, NSE, SCC-AG, CYFRA21-1, and CA199, is more effective than their single detection [7, 8].

In a previous study, epigenetic changes were shown to be significantly associated with NSCLC [9]. Moreover, genes with aberrant DNA methylation were associated with the diagnosis of cancer and treatment prediction and prognostication [10–13], and aberrant DNA methylation was present in the early tumor stage of many cancer types [11, 12]. In addition, aberrantly methylated DNA was found in different types of samples, such as plasma, urine, semen, and stool, indicating that DNA methylation had the potential to become a non-invasive diagnostic biomarker which may facilitate the early diagnosis of NSCLC [11, 14, 15].

However, the value of the detection of gene methylation in blood samples as a non-invasive biomarker in NSCLC remains to be elucidated. Therefore, in our analysis, we aimed to establish a list of genes with altered methylation in NSCLC in an attempt to provide molecular clues for their use as potential biomarkers.

## RESULTS

### Study characteristics

**Figure 1:**
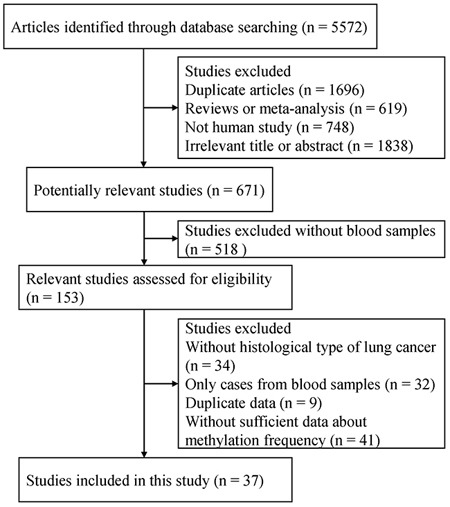
Flow chart of study selection procedure

A total of 5,572 articles were initially identified by a search of PubMed, EMBASE, CNKI and Wanfang literature databases. Among the retrieved the titles, abstracts, and full-text papers, a total of 37 case-control studies published finally met the inclusion criteria based on the use of blood samples in NSCLC. We determined the correlation between aberrantly methylated genes in blood samples and the risk of NSCLC. All eligible studies met a score of equal to or above 5. The process of study selection is summarized in Figure 1, and the detailed characteristics of the included studies are listed in Supplementary Table 1.

### Association between aberrantly methylated genes in the blood and NSCLC

For the analyses of more than three studies on the methylation of *P16*, *DAPK*, and *MGMT* (*I^2^* < 50%, *P* ≥ 0.1), the fixed-effects model was used. The random effects models were applied for methylated *RASSF1A*, *APC*, *RARβ*, *CDH13*, and *FHIT* (*I^2^* > 50%, *P* < 0.1).

**Figure 2:**
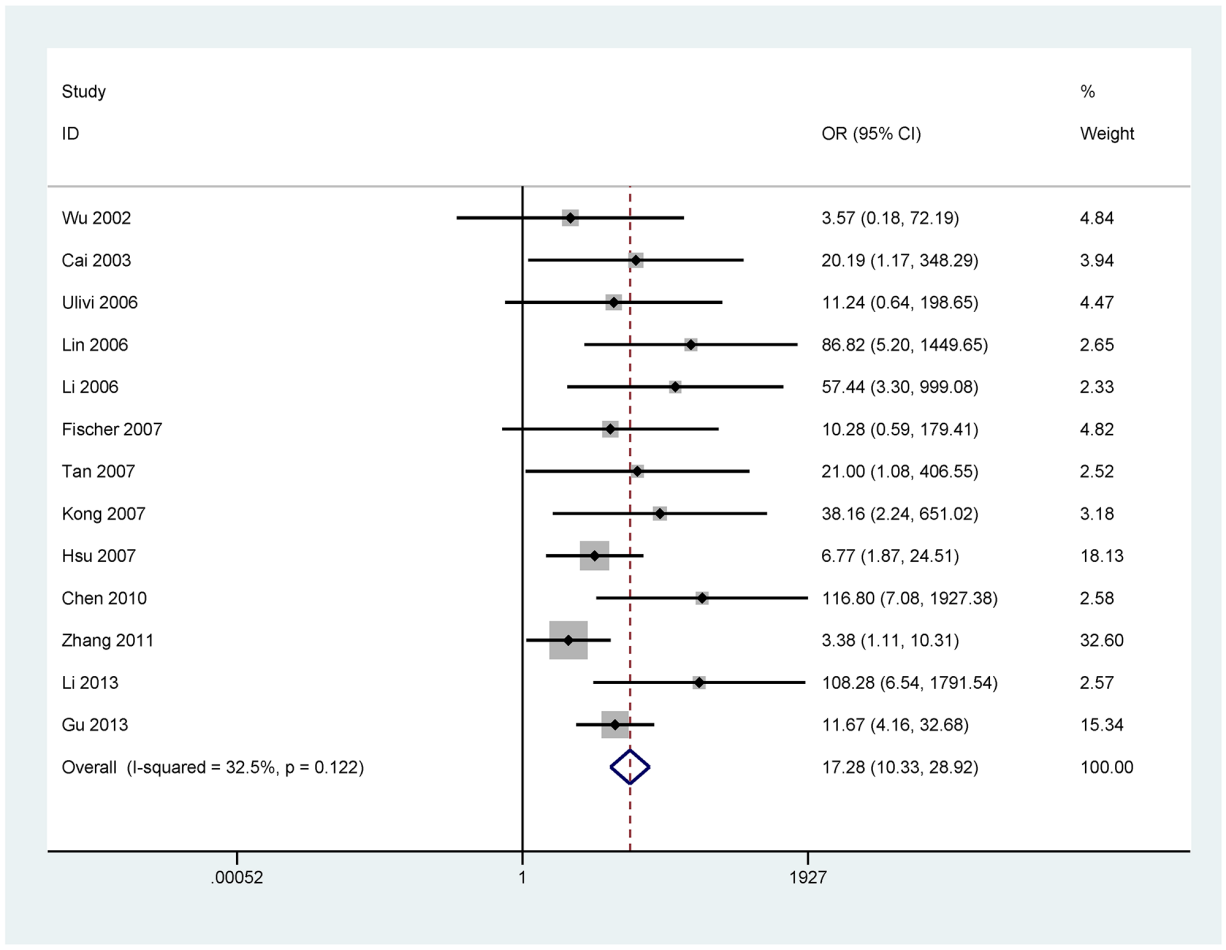
Forest plot of the association between *P16* methylation and NSCLC

**Figure 3:**
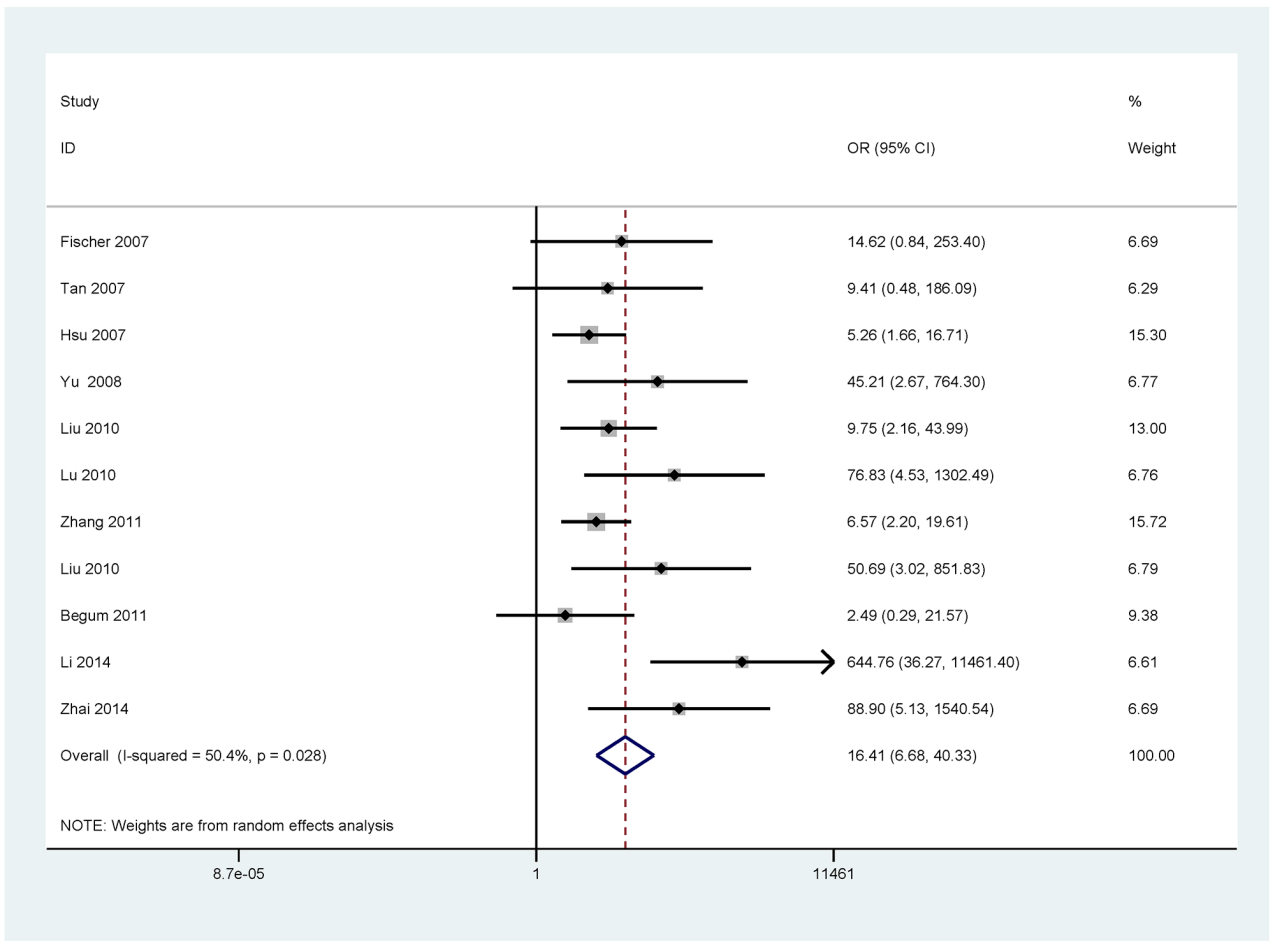
Forest plot of the association between *RASSF1A* methylation and NSCLC

**Figure 4:**
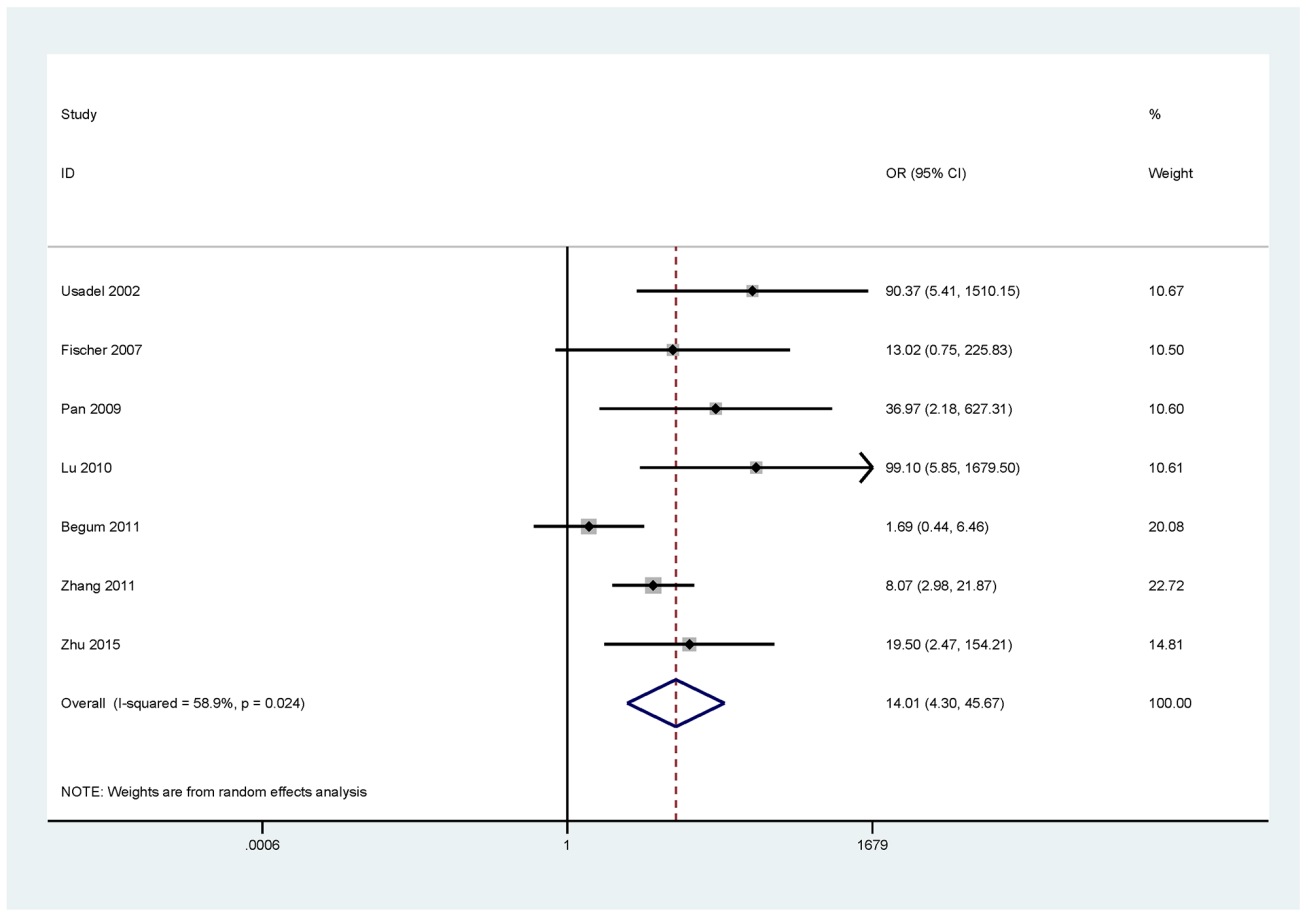
Forest plot of the association between *APC* methylation and NSCLC

**Figure 5:**
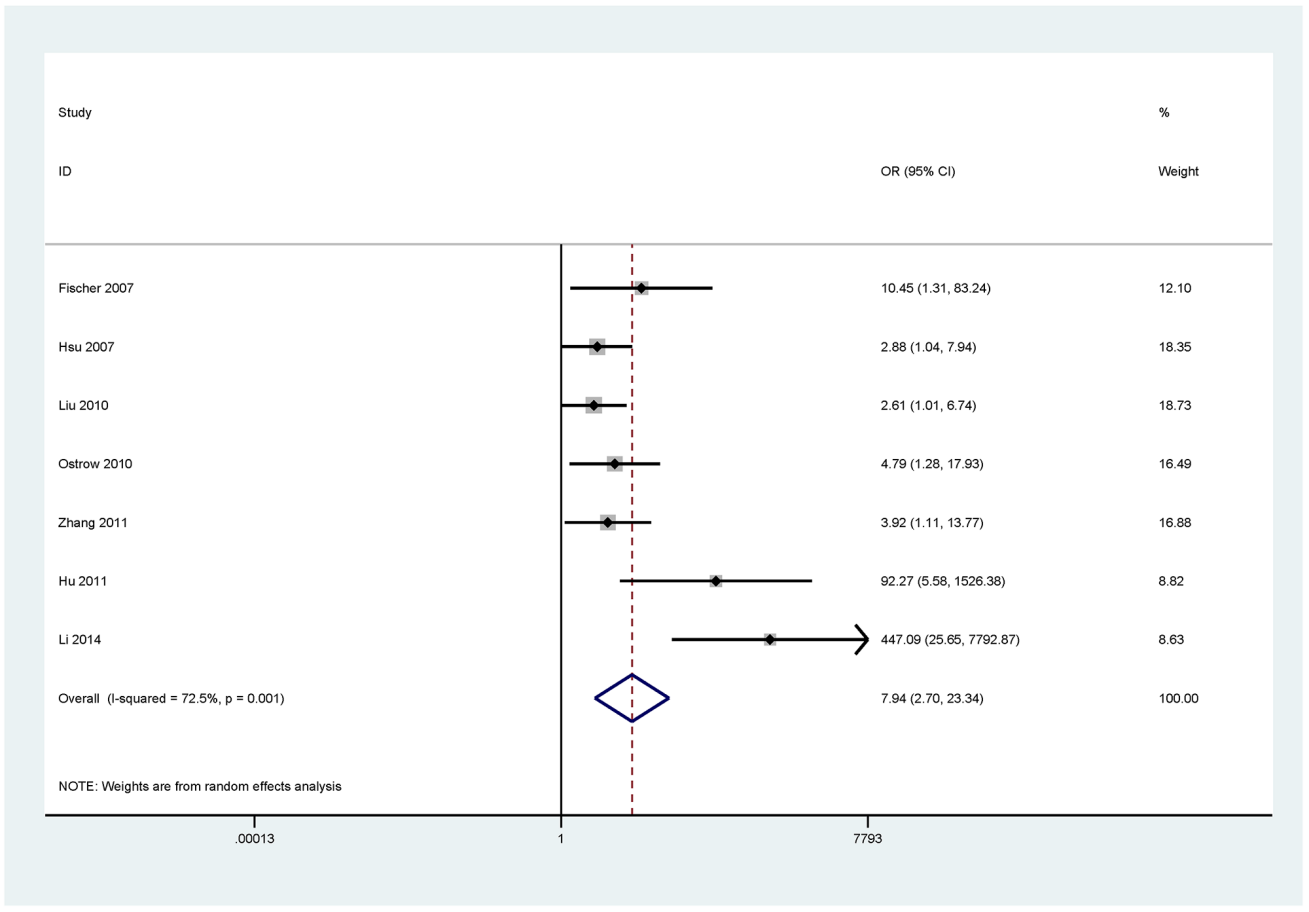
Forest plot of the association between *RARβ* methylation and NSCLC

**Figure 6:**
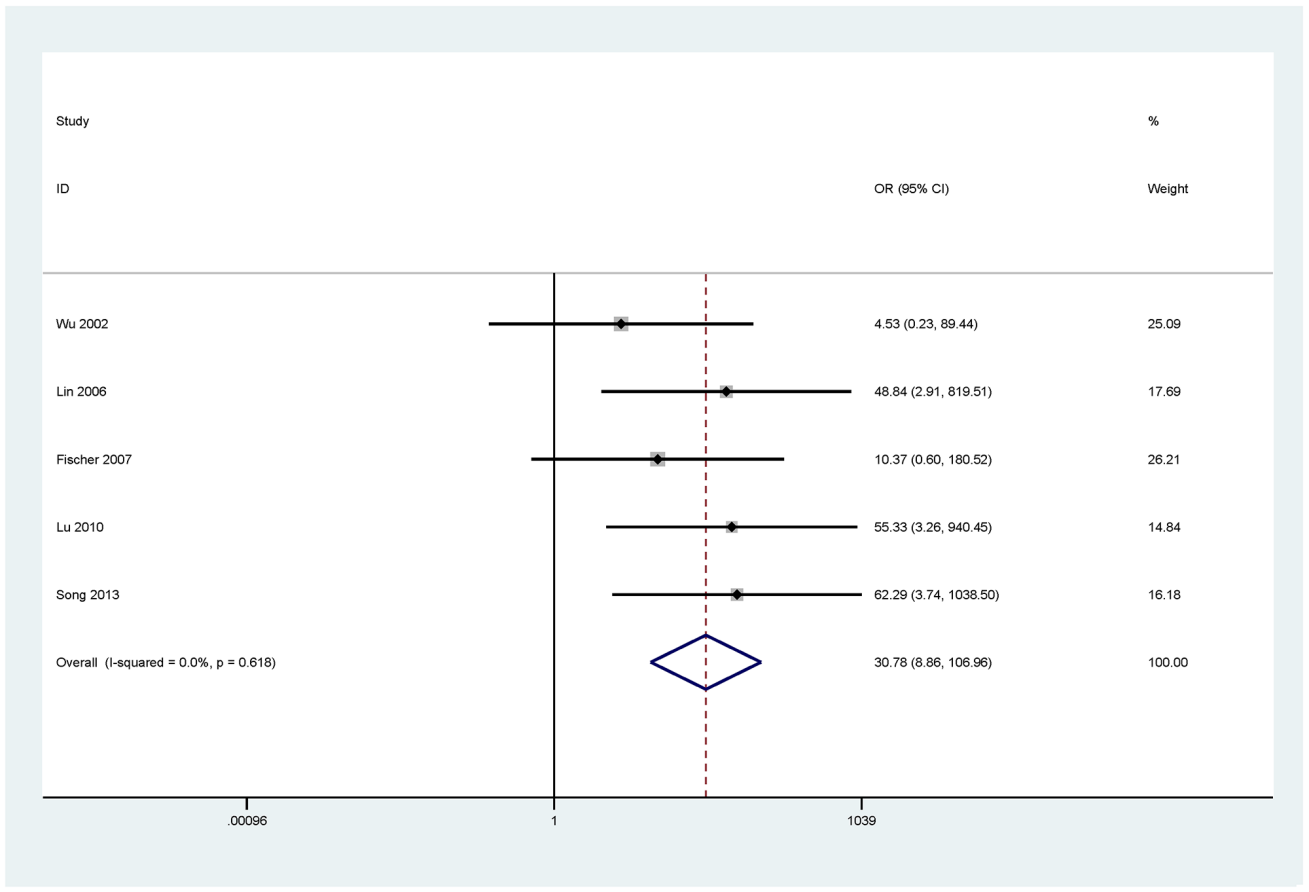
Forest plot of the association between *DAPK* methylation and NSCLC

**Figure 7:**
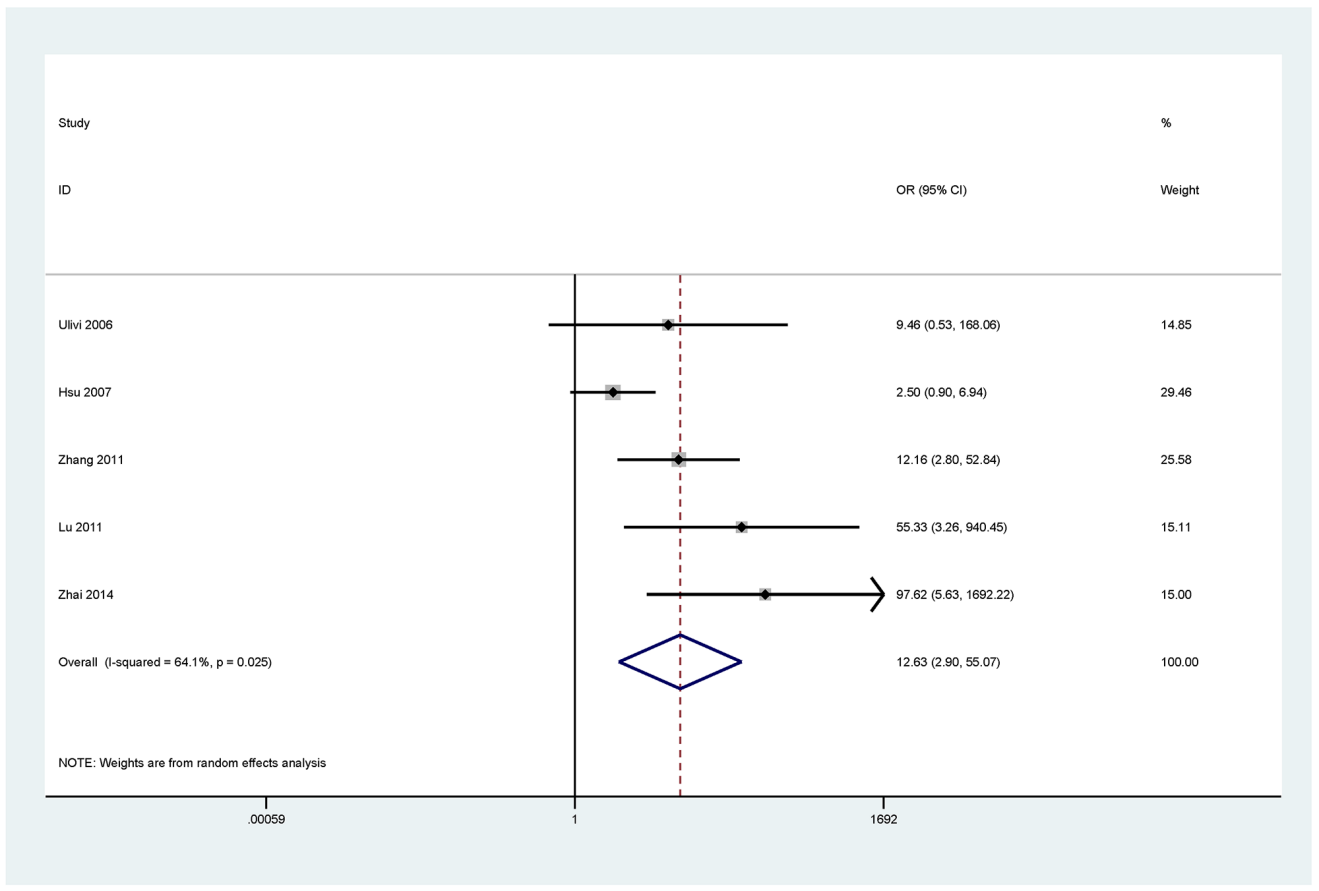
Forest plot of the association between *CDH13* methylation and NSCLC

**Figure 8:**
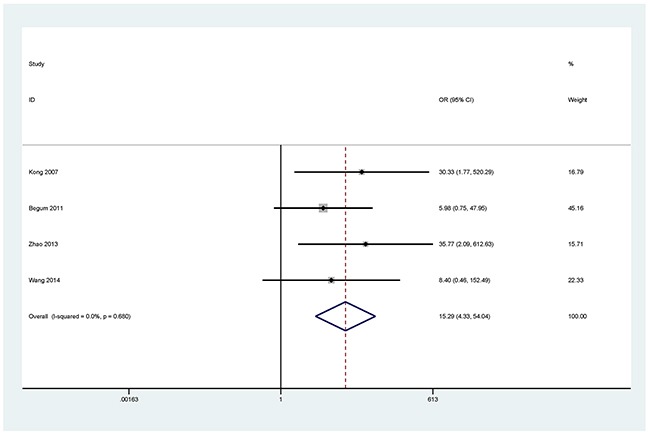
Forest plot of the association between *MGMT* methylation and NSCLC

The results showed that the methylated *P16* (OR = 17.28, *P* < 0.001), *RASSF1A* (OR = 16.41, *P* < 0.001), *APC* (OR = 14.01, *P* < 0.001), *RARβ* (OR = 7.94, *P* < 0.001), *DAPK* (OR = 30.78, *P* < 0.001), *CDH13* (OR = 12.63, *P* = 0.001) and *MGMT* (OR = 15.29, *P* < 0.001) genes were significantly associated with NSCLC in the blood samples (Figures 2, 3, 4, 5, 6, 7, 8). No significant association involving 290 patients with NSCLC and 186 controls was found between *FHIT* methylation and NSCLC (*P* = 0.073) (Table 1).

**Table 1:** Associations between 28 methylated genes detected in blood samples and NSCLC

Gene	Studies	Overall OR (95% CI)	*I^2^*; *P*	*P*-value	Cases	Controls	*P* (Egger's test)
*P16*	13	17.28 (10.33–28.92)	32.5%; 0.122	< 0.001	903	587	0.018
*RASSF1A*	11	16.41 (6.68–40.33)	50.4%; 0.028	< 0.001	770	444	0.016
*APC*	7	14.01 (4.30–45.67)	58.9%; 0.024	< 0.001	563	314	0.088
*RARB*	7	7.94 (2.70–23.34)	72.5%; 0.001	< 0.001	591	436	0.001
*DAPK*	5	30.78 (8.86–106.96)	0.0%; 0.618	< 0.001	385	237	NA
*CDH13*	5	12.63 (2.90–55.07)	64.1%; 0.025	0.001	338	187	NA
*FHIT*	4	4.23 (0.87–20.49)	79.3%; 0.002	0.073	290	186	NA
*MGMT*	4	15.29 ( 4.33–54.04)	0.0%; 0.680	< 0.001	267	129	NA
*DCC*	3	11.44 ( 5.09 - 25.71)	0.0%; 0.531	< 0.001	205	180	NA
*P14*	2	8.95 (1.70–47.19)	0.0%; 0.738	0.01	199	34	NA
*CDH1*	2	3.97 (1.66–9.46)	0.0%; 0.795	0.002	96	40	NA
*RUNX3*	2	45.64 (5.89–353.72)	0.0%; 0.654	< 0.001	82	56	NA
*BLU*	2	1.63 (0.95–2.78)	12.1%; 0.286	0.073	143	116	NA
*SFRP1*	1	7.43 (1.69–32.68)	NA; NA	0.008	110	50	NA
*TMS1*	1	27.80 (1.61–479.59)	NA; NA	0.022	62	46	NA
*TIMP3*	1	35.87 (2.12–607.48)	NA; NA	0.013	110	110	NA
*DLEC1*	1	16.73 (2.21–126.86)	NA; NA	0.006	110	50	NA
*EFEMP1*	1	4.37 (1.25–15.29)	NA; NA	0.021	110	50	NA
*Dkk3*	1	27.43 (7.93–94.86)	NA; NA	< 0.001	75	75	NA
*BRMS1*	1	45.16 (2.60–784.96)	NA; NA	0.009	48	24	NA
*KLK10*	1	9.85 (2.26–42.96)	NA; NA	0.002	110	50	NA
*RASSF2*	1	59.18 (3.48–1005.20)	NA; NA	0.005	62	46	NA
*DCLK1*	1	6.991 (2.74–17.82)	NA; NA	< 0.001	46	95	NA
*AIM1*	1	6.55 (0.82 - 52.21)	NA; NA	0.076	76	30	NA
*hOGG1*	1	3.58 (1.34–9.58)	NA; NA	0.011	80	80	NA
*KIF1A*	1	18.02 (2.29–141.69)	NA; NA	0.006	70	80	NA
*NISCH*	1	2.12 (1.06–4.25)	NA; NA	0.034	70	80	NA
*SEMA3B*	1	11.53 (4.73–28.10)	NA; NA	< 0.001	80	80	NA

Abbreviations: NA: not applicable.

For the remaining 20 genes investigated in less than four studies, 18 genes were shown to be correlated with NSCLC (Table 1), more studies are needed to confirm these results of gene methylation with fewer four studies in the future.

### Subgroup analyses

Subgroup analyses of the methylated *P16*, *RASSF1A*, *APC*, and *RARβ* were performed by methylation detection methods and ethnic population (Caucasians and Asians) (Table 2).

**Table 2:** Subgroup analyses of the associations between *P16*, *RASSF1A*, *APC*, and *RARβ* genes and NSCLC

Gene	Studies	Overall OR 95% CI	*I^2^*; *P*	*P* value	Cases	Controls
*P16*						
Method						
MSP	10	13.09 (5.77–29.71)	29.0%; 0.178	< 0.001	655	386
nMSP	2	67.21 ( 9.15–493.61)	0.0%; 0.578	< 0.001	185	165
qMSP	1	6.77 (1.87–24.51)	NA; NA	0.004	63	36
Race						
Caucasians	2	14.28 (6.93–29.42)	0.0%; 0.965	0.022	146	29
Asians	11	15.92 (6.88–36.83)	44.5%; 0.055	< 0.001	757	558
*RASSF1A*						
Method						
MSP	7	22.17 (6.77–72.60)	50.9%; 0.057	< 0.001	473	300
nMSP	2	12.00 (1.12–129.03)	61.8%; 0.105	0.04	159	68
qMSP	1	2.49 (0.29–21.57)	NA; NA	0.409	76	30
PCR	1	76.83 (4.53–1302.49)	NA; NA	0.003	62	46
Race						
Caucasians	2	4.77 (0.83–27.50)	2.8%; 0.310	0.08	166	44
Asians	9	21.15 (7.62–58.71)	55.6%; 0.021	< 0.001	604	400
*APC*						
Method						
MSP	3	10.32 (4.38–24.32)	0.0%; 0.732	< 0.001	260	134
qMSP	3	14.85 (0.68–322.24)	81.4%; 0.005	0.086	241	134
PCR	1	99.10 (5.85–1679.50)	NA; NA	0.001	62	46
Race						
Caucasians	3	10.44 (0.62–174.92)	77.7%; 0.011	0.103	256	94
Asians	4	17.17 (7.79–37.87)	24.5%; 0.264	< 0.001	307	220
*RARβ*						
Method						
MSP	4	10.02 (1.75–57.37)	79.5%; 0.002	0.01	338	200
nMSP	2	13.65 (0. –805.76)	86.8%; 0.006	0.209	183	156
qMSP	1	4.79 (1.28–17.93)	NA; NA	0.02	70	80
Race						
Caucasians	2	5.99 (1.97–18.27)	0.0%; 0.526	0.002	162	94
Asians	5	7.94 (2.70–23.34)	81.7%; < 0.001	0.003	429	342

Abbreviations: NA: not applicable; MSP: methylation specific PCR; PCR: polymerase chain reaction; qMSP; quantitative methylation specific PCR; nMSP: nested methylation specific PCR.

*P16* methylation was found to be significantly correlated with NSCLC in the MSP, nMSP, and qMSP subgroups (all *P* < 0.01). On the other hand, the subgroup analysis by ethnicity indicated that the methylation of the *P16* gene was significantly associated with NSCLC in Caucasians and Asians (*P* < 0.05).

Varying OR values of *RASSF1A* methylation were obtained in the subgroups of the different methods (MSP: OR = 22.17, *P* < 0.001; nMSP: OR = 12.00, *P* = 0.04; qMSP: *P* = 0.409; PCR: OR = 76.83, *P* = 0.003). It is noteworthy that the association between *RASSF1A* methylation and NSCLC tended to be stronger in Asians (OR = 21.15, *P* < 0.001) than in Caucasians (*P* = 0.08).

Based on the subgroup analysis by methods, significant association between *APC* methylation status and NSCLC was found in the MSP and PCR subgroups (OR = 10.32, *P* < 0.001; OR = 99.10, *P* = 0.001; respectively), but not in the qMSP subgroup (*P* = 0.086). The further subgroup analysis by ethnicity indicated that *APC* methylation was significantly associated with NSCLC in the Asian population (OR = 17.17, *P* < 0.001), but not in Caucasian population (*P* = 0.103).

A statistically significant relationship was found between *RARβ* methylation status and NSCLC in the MSP and qMSP method subgroups (OR = 10.02, *P* = 0.01; OR = 4.79, *P* = 0.02; respectively), but not for the nMSP method (*P* = 0.209). The subgroup analysis by ethnicity revealed that the methylation status of *RARβ* was significantly associated with NSCLC in both the Asian and Caucasian populations (OR = 7.94, *P* = 0.003; OR = 5.99, *P* = 0.002; respectively).

### Meta-regression

Considering the evidence of heterogeneity in the meta-analysis of *RASSF1A* methylation reported in 11 studies (*I^2^* = 50.4%, *P* = 0.028), meta-regression analyses were performed to detect the potential sources of heterogeneity in the methylation detection methods, ethnicity (Caucasians and Asians), and age status (60 or more years: elderly patients; 60 or less years: young patients) (Table 3). The testing method and ethnicity could not explain the sources of heterogeneity (*P* > 0.1); however, the age factor might have been a possible source of heterogeneity (*P* = 0.025).

**Table 3:** Meta-regression analysis of the *RASSF1A* methylation

Subgroup	Coefficient (95% CI)	*t*	*P-*value
Method	-0.901 (-2.467, 0.665)	-1.33	0.221
Ethnicity	0.441 (-2.727, 3.610)	0.32	0.756
Age	2.752 (0.458, 5.046)	2.84	0.025

### Sensitivity analyses

Sensitivity analyses were conducted to assess the stability of the overall effects and the change of heterogeneity by omitting a single study in the meta-analysis of the methylated *RASSF1A*,*APC*, *RARβ*, and *CDH13* (Supplementary Figure 1). The heterogeneity of the *RASSF1A* methylation status was significantly decreased by deleting a single study by Li *et al*. (2014), which caused a change of the *P-*value of the heterogeneity from 0.028 to 0.258. The pooled OR did not substantially change, with a range from 16.41 (95% CI = 6.68– 40.33) to 13.14 (95% CI = 7.55–22.86). The omission of another individual study (Begum *et al*., 2011) increased the *P-*value of heterogeneity of the *APC* methylation status from 0.024 to 0.310, with a rise in the pooled OR from 14.01 (95% CI = 4.30– 45.67) to 20.88 (95% CI = 10.04–43.44). The heterogeneity of the methylated *RARβ* was significantly decreased by omitting a single study by Li *et al*. (2014), with a change of *P-*value of the heterogeneity from 0.001 to 0.129. The overall OR did not significantly change, ranging from 7.94 (95% CI = 2.70– 23.34) to 5.52 (95% CI = 3.37– 9.04). When the study of Hsu*et al*, (2007) was excluded, the pooled OR remained significant, with a range from 12.63 (95% CI = 2.90– 55.07) to 23.30 (95% CI = 8.03– 67.65), and a change of the respective *P-*value of the heterogeneity from 0.025 to 0.483 was observed.

The sensitivity analysis suggested that our results for methylated *RASSF1A*, *APC*, *RARβ*, and *CDH13* were stable.

### Publication bias

Egger's test was performed to estimate the possible publication bias for the methylated *P16*, *RASSF1A*, *APC*, and *RARβ* genes investigated in more than five studies (Supplementary Figure 2). The results of the Egger's test provided statistical data of funnel plot symmetry, which suggested the absence of publication bias concerning the *APC* methylation (*P* = 0.088). There was evidence of publication bias for the methylation status of *P16*, *RASSF1A*, and *RARβ* (*P* < 0.05). Further, we removed one study or two studies to reevaluate whether the potential publication bias for the methylated *P16* and *RASSF1A* genes was reduced. Our results showed that the pooled results regarding *P16* and *RASSF1A* genes did not sustainably change, and no obvious evidence of publication bias was present (Supplementary Figure 3). We further assessed the change of the publication bias by deleting one study or two studies on the *P16* and *RASSF1A* genes. The pooled results of the *P16* and *RASSF1A* genes remained statistically significant, with no substantial evidence of publication bias, indicating credibility. However, a reason for the potential publication bias in the analysis of the low number of studies and/or small sample sizes might have been the unequal comparison of the sample sizes of cases and controls. Thus, we attempted to minimize the bias by using the above-mentioned databases as comprehensively as possible.

### Relation of methylated *P16*, *RASSF1A*, *APC*, *RARβ*, *DAPK*, *CDH13*, and *MGMT* genes to clinicopathological features of NSCLC

We analyzed whether *P16*, *RASSF1A*, *APC*, *RARβ*, *DAPK*, *CDH13*, and *MGMT* methylation status was correlated with clinicopathological characteristics of NSCLC, including age (≥60 years vs. ≤ 60years), gender (male vs. female), smoking behavior (smoking vs. nonsmoking), tumor stage (stage 0–2 vs. stage 3–4), and tumor histology (SCC vs. AC). As depicted in Supplementary Figure 4, the methylation status of *P16*, *RASSF1A*, *APC*, *RARβ*, *DAPK*, *CDH13*, or *MGMT* was not associated with these clinicopathological features (*P* > 0.05), suggesting that the mentioned genes manifested similar methylation properties in the patients examined.

## DISCUSSION

Tumor-specific DNA methylation can be considered a powerful tool for future cancer detection and diagnosis in blood samples in clinical settings [13, 16, 17]. The present analysis was performed to evaluate the potential value of tumor suppressor genes methylation as a feasible biomarker for the detection and screening of NSCLC in blood samples, more specifically with a focus on eight tumor suppressor genes investigated in more than three studies.

*CDKN2A*, also known as *p16/Ink4a* and *p14/ARF*, is one of the major effectors that participate in the oncogenically induced senescence [18–20]. *RASSF1A* may be associated with the transmission of inhibitory growth signals. It is inactivated in the presence of a tumor, and its methylation was detected in many human cancers [21, 22]. *APC* involves in the cell migration and adhesion, transcriptional activation, and apoptosis [23]. Its expression was found to be associated with colorectal cancer [24]. The retinoic acid receptor-β gene (*RARβ*) is shown to be associated with the embryonal development, cell growth, and differentiation [25, 26]. The silencing of the *RARβ* gene expression may lead to resistance to retinoic acid treatment [27]. *DAPK* encodes a cytoskeletal-associated protein kinase (DAPK) that can have some functions in apoptosis and tumor suppression [28, 29]. The downregulation of *CDH13* was linked to a poorer prognosis in patients with various cancer types, including lung cancer [30]. *CDH13* re-expression can reduce tumor growth by inhibiting cell proliferation and invasiveness [30, 31]. *MGMT* encodes O^6^-methylguanine-DNA methyltransferase (MGMT) that is a DNA damage reversal protein [32]. *MGMT* protect the cell from cancer by removing adducts from the O^6^ position of guanine [33]. Some previous studies have shown that some genes with methylation status can become useful biomarkers for NSCLC diagnosis in the mixed samples [34–36]. However, the usefulness of the detection of gene methylation in blood samples from NSCLC patients as a noninvasive biomarker remains to be elucidated.

Increasing evidence suggests that gene methylation may become a potential diagnostic biomarker in NSCLC (i.e., *P16*, *RASSF1A*, *APC*, *RARβ*, *DAPK* and *CDH13*) [37, 38], and serve as a prognostic biomarker such as *P16* and *RASSF1A* [39, 40]. Our findings demonstrated that the methylation status of *P16*, *RASSF1A*, *APC*, *RARβ*, *DAPK*, *CDH13*, and *MGMT* in the blood was correlated with the availability of NSCLC. In addition, we found that methylated genes with good sensitivity and specificity had differently diagnostic levels for NSCLC, such as *P16* gene (sensitivity: 62.5%, specificity: 87.5%) [41], *RASSF1A* (sensitivity: 85.7%, specificity: 100%) [42], *APC* (sensitivity: 51.6%, specificity: 100%) [43], *RARβ* (sensitivity: 80.4%, specificity: 100%) [42], *DAPK* (sensitivity: 37.1%, specificity: 100%) [44], *CDH13* (sensitivity: 54.8%, specificity: 100%) [45], and *MGMT* (sensitivity: 32.8%, specificity: 100%) [46], which suggested that *RASSF1A* and *RARβ* genes can be better promising noninvasive biomarkers for the clinical detection and screening of NSCLC. Further, we analyzed whether the methylation status of *P16*, *RASSF1A*, *APC*, *RARβ*, *DAPK*, *CDH13*, and *MGMT* there were differences in the characteristics of gene methylation in different tumor stages (early stage vs. advanced stage) and tumor histotypes (SCC vs. AC). The results showed that the methylation features of these genes could not facilitate the distinction between early NSCLC and advanced NSCLC, nor between AC, and SCC. One study reported that *RASSF1A* methylation was correlated with overall survival in blood samples of patients with NSCLC, but other genes (*P16*, *RARβ* and *DAPK*) were not found to be linked to overall survival [38]. Two studies reported that *APC* methylation was not associated with survival in blood samples [38, 47].

The current study revealed that the methylation status of *FHIT* detected in blood samples was not associated with NSCLC. In contrast to a previous meta-analysis conducted by Yan *et al*. [48], this study found that *FHIT* methylation was correlated with NSCLC in tissue samples, but this meta-analysis did not analyze whether *FHIT* methylation was linked to NSCLC in blood samples. Additional studies with larger subjects are necessary to further assess the role of *FHIT* methylation in blood samples of patients with NSCLC.

The subgroup analysis by ethnicity regarding the methylation of *P16*, *RASSF1A*, *APC*, and *RARβ* genes showed that the methylation of the *P16* and *RARβ* genes was associated with the occurrence of NSCLC in the Asian and Caucasian populations with, suggesting that the methylation of *P16* and *RARβ* genes can be used as biomarkers for NSCLC detection in both ethnical groups. Methylated *RASSF1A* and *APC* were correlated with NSCLC in the Asian population, but not in the Caucasian population. Nevertheless, this finding could be due to the small sample sizes used in this analysis, especially for the Caucasian population, and thus more studies with larger sample sizes are needed to confirm that result in blood samples from Caucasians with NSCLC.

Further, subgroup analyses by detection methods of *P16*, *RASSF1A*, *APC*, and *RARβ* methylation were conducted. Association between NSCLC and the MSP method was found, suggesting that MSP (n ≥ 3 studies per gene) was a sensitive method for detection of methylated *P16*, *RASSF1A*, *APC*, and *RARβ*. The qMSP method had sensitivity for the detection of *P16* and *RARβ*, but not for *RASSF1A* and *APC*. On the other hand, the nMSP method was sensitive to identification of *P16* and *RASSF1A* methylation, but not to that of *RARβ*. In addition, we found that PCR was sensitive to the methylation of *RASSF1A* and *APC*. However, the findings of the subgroup analyses for qMSP, nMSP, and PCR should be interpreted cautiously due to the small sample sizes of the studies included herein.

The meta-regression analysis of *RASSF1A* methylation revealed that testing method and ethnicity failed to the sources of heterogeneity, but age factor can be a potential source. Sensitivity analyses were also conducted for the *RASSF1A*, *APC*, *RARβ*, and *CDH13* genes with significant heterogeneity. The data based on the omission of one study indicated that the pooled results for the genes investigated remained statistically significant, with absence of heterogeneity, confirming the stability of our findings.

Full-text papers with eligible studies published in English or Chinese were included in this analysis. Nonetheless, investigation in other languages and of other types, such as unpublished studies and conference abstracts, were excluded due to the insufficient availability of information. In addition, studies with positive results are more often and more easily published than those with negative results, which might have contributed to the omission of some examinations. Therefore, in the present meta-analyses, publication bias was detected for the genes investigated in fewer studies and/or such with small sample sizes. Further large-scale studies and well-matched research design with equal comparisons between cases and controls are required in the future to confirm the role of gene methylation as a noninvasive biomarker for NSCLC detection and screening in blood samples, especially for *FHIT*.

Some other limitations of the present research should be carefully considered. First, owing to the limited number of studies with obvious heterogeneity, meta-regression analysis could not be performed for the methylated *APC* and *RARβ*. Moreover, meta-regression and subgroup analyses were not conducted for *CDH13* methylation. Second, the results of some genes methylation and a part of the subgroup analyses should be interpreted with caution as only one or two studies with small subjects were included in our analysis. Finally, gene methylation is shown to be correlated with cigarette smoking factor in mixed samples of patients with NSCLC, including *CDKN2A*, *RASSF1*, *MGMT*, *RARB*, *DAPK*, *WIF1* and *FHIT* [35]. Based on the small sample sizes, our study showed that theses methylated genes was not correlated with smoking behavior in the blood. More large-scale studies are essential to further validate our findings in blood samples of patients with NSCLC.

In conclusion, the use of the *P16*, *RASSF1A*, *APC*, *RARβ*, *DAPK*, *CDH13*, and *MGMT* methylation are exceedingly promising and could become useful biomarkers for blood-based screening and detection of NSCLC in the clinical practice. More studies with larger numbers of blood samples are required to further confirm the diagnostic and screening value of gene methylation in NSCLC.

## MATERIALS AND METHODS

### Literature selection

A comprehensive search was performed in PubMed, EMBASE, CNKI and Wanfang literature databases up to November 26, 2015, using the following search keywords and terms: (lung cancer OR lung tumor OR lung carcinoma OR lung neoplasm OR pulmonary carcinoma) AND (methylation OR epigenetic silencing). No language restriction was employed, and the titles and abstracts were independently assessed by two authors.

The eligible studies had to meet the following criteria: (1) NSCLC patients had to be diagnosed by histopathological examination; (2) gene methylation was evaluated in blood samples in case-control studies, and the control blood samples were collected from individuals with no history of cancer or from healthy subjects; (3) the frequency of gene methylation had to be sufficient to evaluate the associations between gene methylation and NSCLC; (4) the studies were published in English or Chinese. In the case of the presence of more than one published article that had used the same sample data, only the most recent paper or the publication with the larger sample size was selected. Only studies containing data on the histological type of lung cancer were included for inclusion in the current analysis.

### Data extraction and quality assessment

The data extracted from the articles included the following information: the names of the first author, publication year, country, ethnicity, histology, method for the detection of methylation, methylation status. Information on patients’ characteristics was also collected, such as age, gender status, smoking behavior, tumor stage, and pathological subtypes. A cancer stage of ≤ 2 was defined as early, and stages of 3 and 4 were referred to as advanced. The quality assessment of the included studies was performed based on the Newcastle–Ottawa Scale (NOS), with a range from 0 to 9. Each study with a NOS score of more than or equal to six was considered as high quality, and a NOS score of less than or equal to three was considered as low quality [49, 50] (Supplementary Table 1).

### Data analysis

The pooled odds ratio (OR) with the corresponding 95% confidence interval (95% CI) was calculated using Stata software (STATA version 12.0, Stata Corporation, College Station, TX, USA) to evaluate the relationships between gene methylation and risk of NSCLC. The statistical heterogeneity among the studies included in the meta-analysis was assessed by Cochran's Q statistic and *I^2^* test [51]. The fixed-effect model was applied for the meta-analysis with moderate or lack of heterogeneity (*I^2^* < 50% and *P* ≥ 0.1); otherwise, the random-effects model was employed [52, 53]. Meta-regression analysis was used to identify the potential sources of heterogeneity. Subgroup analyses were conducted to find the variations among the different subgroups. Sensitivity analysis was also performed by omitting one study to assess the influence of an individual study on the overall OR [54]. *P* < 0.05 was considered significant. Egger's test was used to estimate the potential publication bias for the methylated genes investigated in more than five studies [55].

## SUPPLEMENTARY MATERIALS FIGURES AND TABLES




