# Supplementary material for: Gene methylation as a powerful biomarker for detection and screening of non-small cell lung cancer in blood
Source: Oncotarget. 2017 Mar 6;8(19):31692–704. doi: 10.18632/oncotarget.15919 (PMC5458240; doi:10.18632/oncotarget.15919)
Supplement: Supplementary file 1 [file oncotarget-08-31692-s001.pdf]

Gene methylation as a powerful biomarker for detection and screening of non-small cell lung cancer in blood

SUPPLEMENTARY MATERIALS

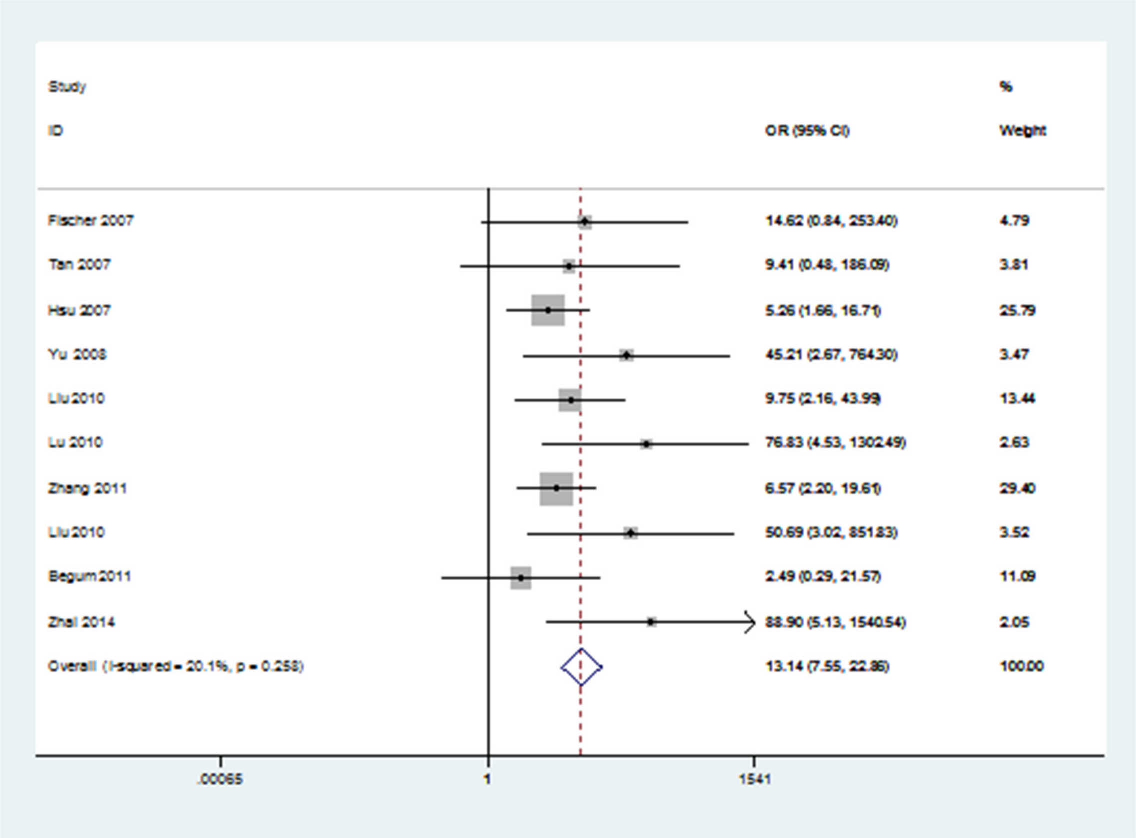

Supplementary Figure 1: The sensitivity analyses by omitting a single study in the meta-analysis of the methylated *RASSF1A*, *APC*, *RARβ*, and *CDH13*.

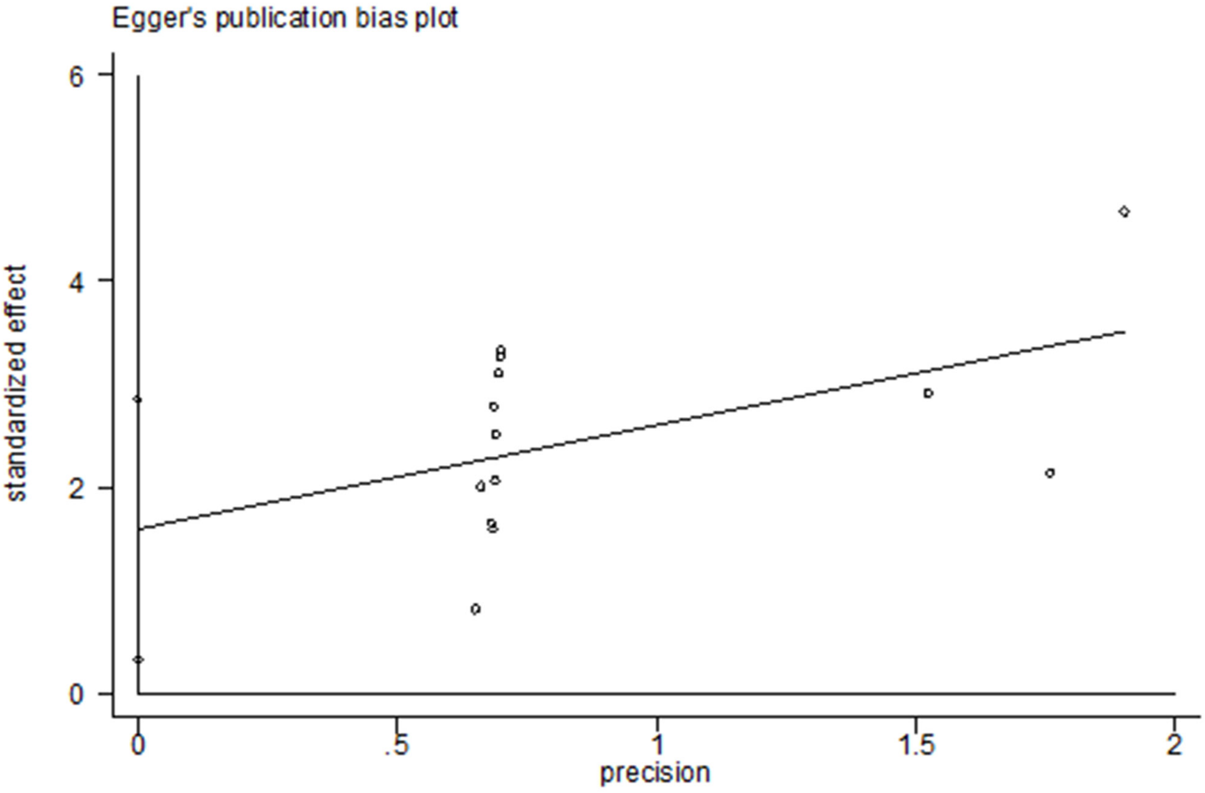

Supplementary Figure 2: Egger's funnel plots of the publication bias for methylated *P16*, *RASSF1A*, *APC*, and *RARβ* genes investigated in more than five studies.

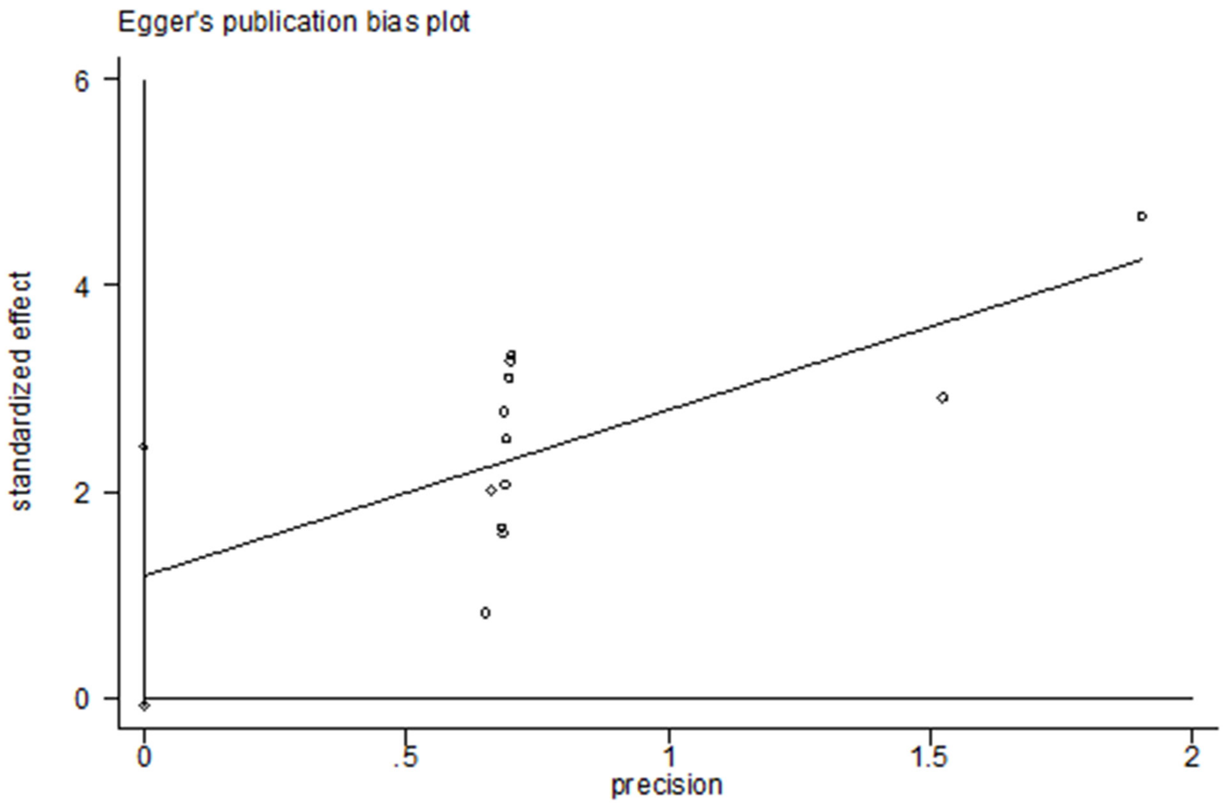

Supplementary Figure 3: Egger's funnel plots of the publication bias and forest plots of the correlation for the methylated *P16* and *RASSF1A* genes conducted after omitting one or two studies.

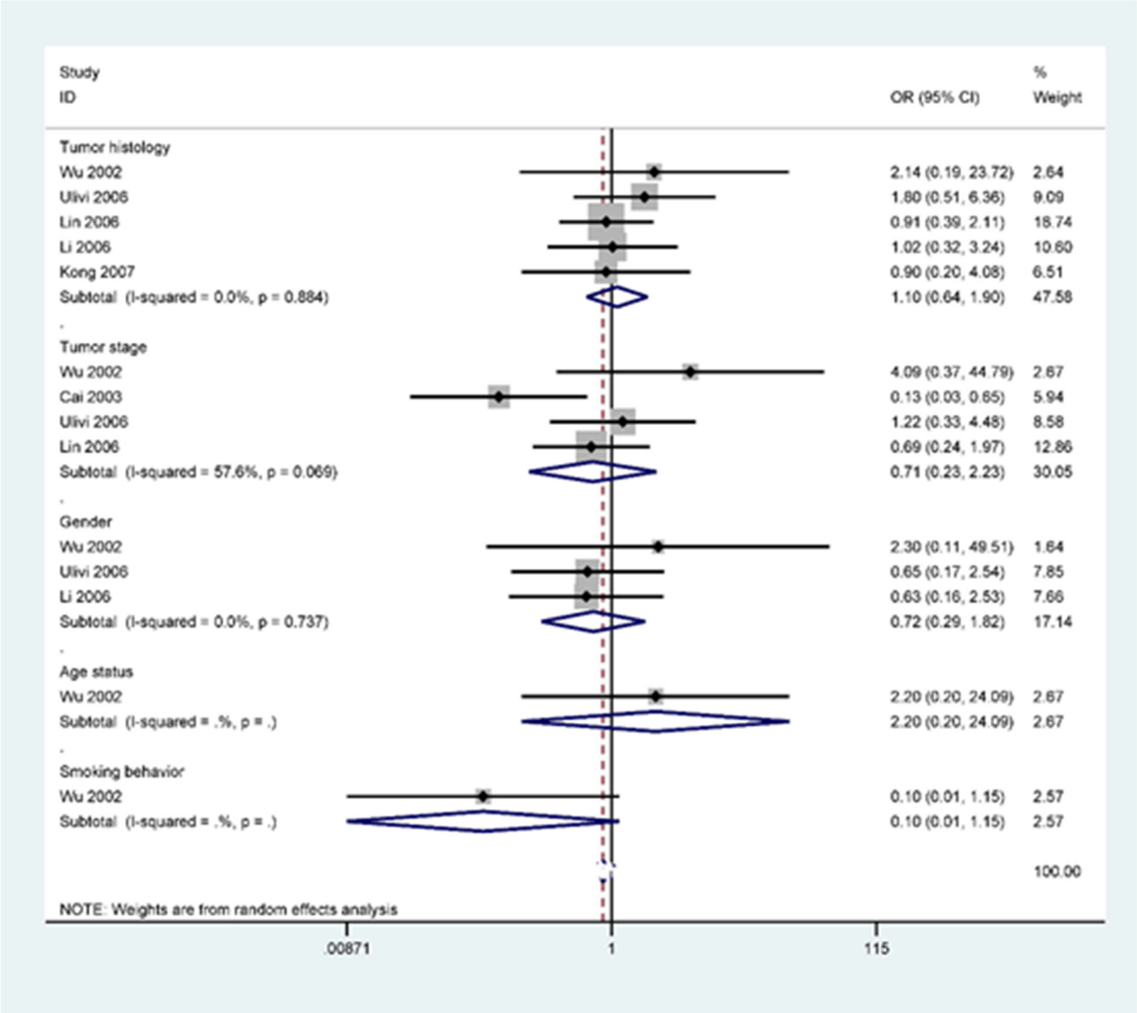

Supplementary Figure 4: Forest plots of the correlation between methylated *P16*, *RASSF1A*, *APC*, *RARβ*, *DAPK*, *CDH13*, and *MGMT* genes and clinicopathological features.

Supplementary Table 1: General characteristics of the included studies based on the comparison of blood samples from NSCLC patients vs. controls.

See Supplementary File 1
