# Supplementary material for: Gene methylation as a powerful biomarker for detection and screening of non-small cell lung cancer in blood
Source: Oncotarget. 2017 Mar 6;8(19):31692–704. doi: 10.18632/oncotarget.15919 (PMC5458240; doi:10.18632/oncotarget.15919)
Supplement: Supplementary file 2 [file oncotarget-08-31692-s002.docx]

Table S1. General characteristics of the included studies based on the comparison of blood samples from NSCLC patients vs. controls.

| Gene | First author | Age | Stage | SCC/AC | Male/Female | Smoking | Country | Ethnicity | Method | Case | Control | > = 60 years | < 60 years | Smoking | Nonsmoking | Male | Female | Stage 0-2 | Stage 3-4 | SCC | AC | NOS |
| --- | --- | --- | --- | --- | --- | --- | --- | --- | --- | --- | --- | --- | --- | --- | --- | --- | --- | --- | --- | --- | --- | --- |
|  |  |  |  | Patients | Patients | Patients |  |  |  | M+/N | M+/N | M+/N | M+/N | M+/N | M+/N | M+/N | M+/N | M+/N | M+/N | M+/N | M+/N |  |
| *P16* |  |  |  |  |  |  |  |  |  |  |  |  |  |  |  |  |  |  |  |  |  |  |
|  | Wu 2002 | 41-76 | 1-3 | 17/11 | 25/5 | 21 | China | Asians | MSP | 4/30 | 0/10 | 3/18 | 1/12 | 1/21 | 3/9 | 4/25 | 0/5 | 3/14 | 1/16 | 3/17 | 1/11 | 8 |
|  | Cai 2003 | 59.5 | 1-4 | 25/36 | 46/23 | NA | China | Asians | MSP | 15/69 | 0/35 |  |  |  |  |  |  | 2/31 | 13/38 |  |  | 6 |
|  | Ulivi 2006 | 70 | 1/3 | 16/36 | 49/12 | NA | Italy | Caucasians | MSP | 16/61 | 0/15 |  |  |  |  | 12/49 | 4/12 | 12/44 | 4/17 | 6/16 | 9/36 | 6 |
|  | Fischer 2007 | 60.9 | 3-4 | 28/48 | 60/32 | NA | Germany | Caucasians | MSP | 22/85 | 0/14 |  |  |  |  |  |  |  |  |  |  | 6 |
|  | Lin 2006 | 65 | 1/4 | 40/49 | 69/30 | NA | China | Asians | MSP | 39/89 | 0/55 |  |  |  |  |  |  | 7/19 | 32/70 | 17/40 | 22/49 | 6 |
|  | Li 2006 | NA | NA | 22/24 | 38/11 | NA | China | Asians | MSP | 27/49 | 0/23 |  |  |  |  | 20/38 | 7/11 |  |  | 12/22 | 13/24 | 7 |
|  | Tan 2007 | NA | NA | NA | NA | NA | Singapore | Asians | MSP | 10/20 | 0/10 |  |  |  |  |  |  |  |  |  |  | 5 |
|  | Zhang 2011 | NA | 1-2 | NA | NA | NA | China | Asians | MSP | 25/110 | 4/50 |  |  |  |  |  |  |  |  |  |  | 7 |
|  | Li 2013 | 59. 65 | 0-4 | 61/24 | 94/16 | NA | China | Asians | MSP | 36/110 | 0/110 |  |  |  |  |  |  |  |  |  |  | 8 |
|  | Gu 2013 | 47.5 | 1 | 11/17 | 18/14 | 11 | China | Asians | MSP | 20/32 | 8/64 |  |  |  |  |  |  |  |  |  |  | 8 |
|  | Kong 2007 | 58.7 | 1-3 | 43/10 | 60/5 | NA | China | Asians | nMSP | 19/65 | 0/45 |  |  |  |  |  |  |  |  | 12/43 | 3/10 | 6 |
|  | Chen 2010 | 59.65 | 0-4 | 66/26 | 102/18 | NA | China | Asians | nMSP | 39/120 | 0/120 |  |  |  |  |  |  |  |  |  |  | 6 |
|  | Hsu 2007 | 69 | 1-4 | 13/41 | 45/18 | 37 | Taiwan | Asians | qMSP | 24/63 | 3/36 |  |  |  |  |  |  |  |  |  |  | 8 |
|  |  |  |  |  |  |  |  |  |  |  |  |  |  |  |  |  |  |  |  |  |  |  |
| *RASSF1A* | |  |  |  |  |  |  |  |  |  |  |  |  |  |  |  |  |  |  |  |  |  |
|  | Fischer 2007 | 60.9 | 3-4 | 28/48 | 60/32 | NA | Germany | Caucasians | MSP | 30/90 | 0/14 |  |  |  |  |  |  |  |  |  |  | 6 |
|  | Begum 2011 | 65 | 1-4 | 26/36 | 40/36 | NA | USA | Caucasians | qMSP | 6/76 | 1/30 |  |  |  |  |  |  |  |  | 3/26 | 2/36 | 7 |
|  | Tan 2007 | NA | NA | NA | NA | NA | Singapore | Asians | MSP | 6/20 | 0/10 |  |  |  |  |  |  |  |  |  |  | 5 |
|  | Yu 2008 | 62 | 1-4 | 26/40 | 44/31 | NA | China | Asians | MSP | 23/75 | 0/50 | 13/43 | 10/32 |  |  | 13/44 | 10/31 | 4/27 | 19/48 | 6/26 | 15/40 | 6 |
|  | Liu 2010 | 61 | 1-4 | 37/22 | 59/21 | 47 | China | Asians | MSP | 16/80 | 2/80 |  |  |  |  |  |  |  |  |  |  | 8 |
|  | Lu 2010 | 59.6 | 1-4 | 27/35 | 40/22 | NA | China | Asians | PCR | 28/62 | 0/46 | 15/39 | 13/23 |  |  | 17/40 | 11/22 | 1/11 | 27/51 | 10/27 | 18/35 | 6 |
|  | Zhang 2011 | NA | 1-2 | NA | NA | NA | China | Asians | MSP | 40/110 | 4/50 |  |  |  |  |  |  |  |  |  |  | 7 |
|  | Hsu 2007 | 69 | 1-4 | 13/41 | 45/18 | 37 | Taiwan | Asians | nMSP | 25/63 | 4/36 |  |  |  |  |  |  |  |  |  |  | 8 |
|  | Liu 2010 | 57 | 1-4 | 42/54 | 54/42 | 48 | China | Asians | nMSP | 42/96 | 0/32 |  |  |  |  |  |  |  |  |  |  | 6 |
|  | Li 2014 | 55.1 | 1-4 | 29/17 | NA | NA | China | Asians | MSP | 48/56 | 0/56 |  |  |  |  |  |  | 20/22 | 28/34 | 26/29 | 14/17 | 7 |
|  | Zhai 2014 | 62.39 | 1-4 | 10/32 | 32/10 | 22 | China | Asians | MSP | 22/42 | 0/40 | 15/29 | 7/13 | 10/22 | 12/20 | 15/32 | 7/10 | 2/6 | 20/36 | 5/10 | 17/32 | 6 |
|  |  |  |  |  |  |  |  |  |  |  |  |  |  |  |  |  |  |  |  |  |  |  |
| *APC* |  |  |  |  |  |  |  |  |  |  |  |  |  |  |  |  |  |  |  |  |  |  |
|  | Usadel 2002 | NA | NA | NA | NA | NA | USA | Caucasians | qMSP | 42/89 | 0/50 |  |  |  |  |  |  |  |  |  |  | 8 |
|  | Fischer 2007 | 60.9 | 3-4 | 28/48 | 60/32 | NA | Germany | Caucasians | MSP | 28/91 | 0/14 |  |  |  |  |  |  |  |  |  |  | 6 |
|  | Pan 2009 | NA | NA | NA | NA | NA | China | Asians | qMSP | 19/76 | 0/54 |  |  |  |  |  |  |  |  |  |  | 6 |
|  | Lu 2010 | 59.6 | 1-4 | 27/35 | 40/22 | NA | China | Asians | PCR | 32/62 | 0/46 | 19/39 | 13/23 |  |  | 22/40 | 10/22 | 7/11 | 25/51 | 10/27 | 22/35 | 6 |
|  | Begum 2011 | 65 | 1-4 | 26/36 | 40/36 | NA | USA | Caucasians | qMSP | 12/76 | 3/30 |  |  |  |  |  |  |  |  | 6/26 | 5/36 | 7 |
|  | Zhang 2011 | NA | 1-2 | NA | NA | NA | China | Asians | MSP | 52/110 | 5/50 |  |  |  |  |  |  |  |  |  |  | 7 |
|  | Zhu 2015 | NA | 1-4 | 30/29 | NA | NA | China | Asians | MSP | 13/59 | 1/70 |  |  |  |  |  |  |  |  | 6/30 | 7/29 | 6 |
|  |  |  |  |  |  |  |  |  |  |  |  |  |  |  |  |  |  |  |  |  |  |  |
| *RARβ* | |  |  |  |  |  |  |  |  |  |  |  |  |  |  |  |  |  |  |  |  |  |
|  | Fischer 2007 | 60.9 | 3-4 | 28/48 | 60/32 | NA | Germany | Caucasians | MSP | 41/92 | 1/14 |  |  |  |  |  |  |  |  |  |  | 6 |
|  | Liu 2010 | 61 | 1-4 | 37/22 | 59/21 | 47 | China | Asians | MSP | 16/80 | 7/80 |  |  |  |  |  |  |  |  |  |  | 8 |
|  | Li 2014 | 55.1 | 1-4 | 29/17 | NA | NA | China | Asians | MSP | 45/56 | 0/56 |  |  |  |  |  |  | 16/22 | 29/34 | 23/29 | 15/17 | 7 |
|  | Zhang 2011 | NA | 1-2 | NA | NA | NA | China | Asians | MSP | 22/110 | 3/50 |  |  |  |  |  |  |  |  |  |  | 7 |
|  | Hsu 2007 | 69 | 1-4 | 13/41 | 45/18 | 37 | Taiwan Wan | Asians | nMSP | 23/63 | 6/36 |  |  |  |  |  |  |  |  |  |  | 8 |
|  | Hu 2011 | 59.65 | 0-4 | 66/26 | 102/18 | NA | China | Asians | nMSP | 33/120 | 0/120 |  |  |  |  |  |  |  |  |  |  | 7 |
|  | Ostrow 2010 | 69 | 1-4 | 7/47 | 37/33 | 68 | USA | Caucasians | qMSP | 11/70 | 3/80 |  |  |  |  |  |  |  |  |  |  | 8 |
|  |  |  |  |  |  |  |  |  |  |  |  |  |  |  |  |  |  |  |  |  |  |  |
| *DAPK* | |  |  |  |  |  |  |  |  |  |  |  |  |  |  |  |  |  |  |  |  |  |
|  | Wu 2002 | 41-76 | 1-3 | 17/11 | 25/5 | 21 | China | Asians | MSP | 5/30 | 0/10 | 2/18 | 3/12 | 4/21 | 1/9 | 4/25 | 1/5 | 1/14 | 4/16 | 3/17 | 1/11 | 8 |
|  | Lin 2006 | 65 | 1/4 | 40/49 | 69/30 | NA | China | Asians | MSP | 27/89 | 0/55 |  |  |  |  |  |  | 4/19 | 23/70 | 10/40 | 17/49 | 6 |
|  | Fischer 2007 | 60.9 | 3-4 | 28/48 | 60/32 | NA | Germany | Caucasians | MSP | 24/92 | 0/14 |  |  |  |  |  |  |  |  |  |  | 6 |
|  | Lu 2010 | 59.6 | 1-4 | 27/35 | 40/22 | NA | China | Asians | PCR | 23/62 | 0/46 | 13/39 | 10/23 |  |  | 15/40 | 8/22 | 3/11 | 20/51 | 7/27 | 16/35 | 6 |
|  | Song 2013 | 61 | 0-4 | 56/27 | 91/21 | NA | China | Asians | nMSP | 24/112 | 0/112 |  |  |  |  |  |  |  |  |  |  | 8 |
|  |  |  |  |  |  |  |  |  |  |  |  |  |  |  |  |  |  |  |  |  |  |  |
| *CDH13* | |  |  |  |  |  |  |  |  |  |  |  |  |  |  |  |  |  |  |  |  |  |
|  | Ulivi 2006 | 70 | 1/3 | 16/36 | 49/12 | NA | Italy | Caucasians | FMSP | 14/61 | 0/15 |  |  |  |  | 12/49 | 2/12 | 9/44 | 5/17 | 4/16 | 9/36 | 6 |
|  | Hsu 2007 | 69 | 1-4 | 13/41 | 45/18 | 37 | Taiwan | Asians | nMSP | 21/63 | 6/36 |  |  |  |  |  |  |  |  |  |  | 8 |
|  | Zhang 2011 | NA | 1-2 | NA | NA | NA | China | Asians | MSP | 37/110 | 2/50 |  |  |  |  |  |  |  |  |  |  | 7 |
|  | Lu 2011 | 62 | 1-4 | 27/35 | 40/22 | NA | China | Asians | MSP | 23/62 | 0/46 | 14/39 | 9/23 |  |  | 13/40 | 10/22 | 8/11 | 17/51 | 7/27 | 18/35 | 7 |
|  | Zhai 2014 | 62.39 | 1-4 | 10/32 | 32/10 | 22 | China | Asians | MSP | 23/42 | 0/40 | 14/29 | 9/13 | 13/22 | 10/20 | 19/32 | 4/10 | 3/6 | 20/36 | 6/10 | 17/32 | 6 |
|  |  |  |  |  |  |  |  |  |  |  |  |  |  |  |  |  |  |  |  |  |  |  |
| *FHIT* |  |  |  |  |  |  |  |  |  |  |  |  |  |  |  |  |  |  |  |  |  |  |
|  | Fischer 2007 | 60.9 | 3-4 | 28/48 | 60/32 | NA | Germany | Caucasians | MSP | 43/91 | 0/14 |  |  |  |  |  |  |  |  |  |  | 6 |
|  | Hsu 2007 | 69 | 1-4 | 13/41 | 45/18 | 37 | Taiwan | Asians | nMSP | 20/63 | 7/36 |  |  |  |  |  |  |  |  |  |  | 8 |
|  | Liu 2010 | 61 | 1-4 | 37/22 | 59/21 | 47 | China | Asians | MSP | 19/80 | 19/80 |  |  |  |  |  |  |  |  |  |  | 8 |
|  | Li 2014 | 55.1 | 1-4 | 29/17 | NA | NA | China | Asians | MSP | 19/56 | 0/56 |  |  |  |  |  |  | 4/22 | 15/34 | 9/29 | 6/17 | 7 |
|  |  |  |  |  |  |  |  |  |  |  |  |  |  |  |  |  |  |  |  |  |  |  |
| *MGMT* | |  |  |  |  |  |  |  |  |  |  |  |  |  |  |  |  |  |  |  |  |  |
|  | Kong 2007 | 58.7 | 1-3 | 43/10 | 60/5 | NA | China | Asians | nMSP | 16/65 | 0/45 |  |  |  |  |  |  |  |  | 11/43 | 2/10 | 6 |
|  | Begum 2011 | 65 | 1-4 | 26/36 | 40/36 | NA | USA | Caucasians | qMSP | 13/76 | 1/30 |  |  |  |  |  |  |  |  | 4/26 | 7/36 | 7 |
|  | Zhao 2013 | 59.6 | 1-4 | 27/35 | 40/22 | NA | China | Asians | PCR | 17/62 | 0/46 |  |  |  |  |  |  | 0/11 | 17/51 |  |  | 7 |
|  | Wang 2014 | 43 | 2-3 | 26/38 | 39/25 | NA | China | Asians | Nmsp | 21/64 | 0/8 |  |  |  |  |  |  |  |  |  |  | 5 |
|  |  |  |  |  |  |  |  |  |  |  |  |  |  |  |  |  |  |  |  |  |  |  |
| *DCC* |  |  |  |  |  |  |  |  |  |  |  |  |  |  |  |  |  |  |  |  |  |  |
|  | Ostrow 2010 | 69 | 1-4 | 7/47 | 37/33 | 68 | USA | Caucasians | qMSP | 19/70 | 4/80 |  |  |  |  |  |  |  |  |  |  | 8 |
|  | Begum 2011 | 65 | 1-4 | 26/36 | 40/36 | NA | USA | Caucasians | qMSP | 27/76 | 0/30 |  |  |  |  |  |  |  |  | 12/26 | 10/36 | 7 |
|  | Zhu 2015 | NA | 1-4 | 30/29 | NA | NA | China | Asians | MSP | 21/59 | 3/70 |  |  |  |  |  |  |  |  | 9/30 | 12/29 | 6 |
|  |  |  |  |  |  |  |  |  |  |  |  |  |  |  |  |  |  |  |  |  |  |  |
| *P14* |  |  |  |  |  |  |  |  |  |  |  |  |  |  |  |  |  |  |  |  |  |  |
|  | Fischer 2007 | 60.9 | 3-4 | 28/48 | 60/32 | NA | Germany | Caucasians | MSP | 28/92 | 0/14 |  |  |  |  |  |  |  |  |  |  | 6 |
|  | Li 2013 | >=29 | 1-3 | 43/56 | 81/26 | 77 | China | Asians | nMSP | 29/107 | 1/20 |  |  |  |  |  |  |  |  |  |  | 5 |
|  |  |  |  |  |  |  |  |  |  |  |  |  |  |  |  |  |  |  |  |  |  |  |
| *CDH1* | |  |  |  |  |  |  |  |  |  |  |  |  |  |  |  |  |  |  |  |  |  |
|  | Tan 2007 | NA | NA | NA | NA | NA | Singapore | Asians | MSP | 4/20 | 0/10 |  |  |  |  |  |  |  |  |  |  | 5 |
|  | Begum 2011 | 65 | 1-4 | 26/36 | 40/36 | NA | USA | Caucasians | qMSP | 47/76 | 9/30 |  |  |  |  |  |  |  |  | 9/26 | 5/36 | 7 |
|  |  |  |  |  |  |  |  |  |  |  |  |  |  |  |  |  |  |  |  |  |  |  |
| *RUNX3* | |  |  |  |  |  |  |  |  |  |  |  |  |  |  |  |  |  |  |  |  |  |
|  | Tan 2007 | NA | NA | NA | NA | NA | Singapore | Asians | MSP | 11/20 | 0/10 |  |  |  |  |  |  |  |  |  |  | 5 |
|  | Lu 2011 | 59.6 | 1-4 | 27/35 | 40/22 | NA | China | Asians | MSP | 25/62 | 0/46 | 15/39 | 10/23 |  |  | 16/40 | 9/22 | 1/11 | 22/51 | 8/27 | 15/35 | 7 |
|  |  |  |  |  |  |  |  |  |  |  |  |  |  |  |  |  |  |  |  |  |  |  |
| *SFRP1* | |  |  |  |  |  |  |  |  |  |  |  |  |  |  |  |  |  |  |  |  |  |
|  | Zhang 2011 | NA | 1-2 | NA | NA | NA | China | Asians | MSP | 26/110 | 2/50 |  |  |  |  |  |  |  |  |  |  | 7 |
|  |  |  |  |  |  |  |  |  |  |  |  |  |  |  |  |  |  |  |  |  |  |  |
| *TMS1/ASC* | |  |  |  |  |  |  |  |  |  |  |  |  |  |  |  |  |  |  |  |  |  |
|  | Lu 2013 | 59.6 | 1-4 | 27/35 | 40/22 | NA | China | Asians | MSP | 14/62 | 0/46 | 9/39 | 5/23 |  |  | 8/40 | 6/22 | 0/11 | 14/51 | 4/27 | 10/35 | 7 |
|  |  |  |  |  |  |  |  |  |  |  |  |  |  |  |  |  |  |  |  |  |  |  |
| *TIMP3* | |  |  |  |  |  |  |  |  |  |  |  |  |  |  |  |  |  |  |  |  |  |
|  | Li 2013 | 59. 65 | 0-4 | 61/24 | 94/16 | NA | China | Asians | MSP | 15/110 | 0/110 |  |  |  |  |  |  |  |  |  |  | 8 |
|  |  |  |  |  |  |  |  |  |  |  |  |  |  |  |  |  |  |  |  |  |  |  |
| *DLEC1* | |  |  |  |  |  |  |  |  |  |  |  |  |  |  |  |  |  |  |  |  |  |
|  | Zhang 2011 | NA | 1-2 | NA | NA | NA | China | Asians | MSP | 28/110 | 1/50 |  |  |  |  |  |  |  |  |  |  | 7 |
|  |  |  |  |  |  |  |  |  |  |  |  |  |  |  |  |  |  |  |  |  |  |  |
| *EFEMP1* | |  |  |  |  |  |  |  |  |  |  |  |  |  |  |  |  |  |  |  |  |  |
|  | Zhang 2011 | NA | 1-2 | NA | NA | NA | China | Asians | MSP | 24/110 | 3/50 |  |  |  |  |  |  |  |  |  |  | 7 |
|  |  |  |  |  |  |  |  |  |  |  |  |  |  |  |  |  |  |  |  |  |  |  |
| *Dkk3* | |  |  |  |  |  |  |  |  |  |  |  |  |  |  |  |  |  |  |  |  |  |
|  | Lei 2013 | 48.2 | 1-4 | 35/28 | 45/30 | 40 | China | Asians | MSP | 40/75 | 3/75 |  |  | 23/40 | 17/35 | 24/45 | 16/30 | 14/37 | 26/38 | 19/35 | 15/28 | 8 |
|  |  |  |  |  |  |  |  |  |  |  |  |  |  |  |  |  |  |  |  |  |  |  |
| *BRMS1* | |  |  |  |  |  |  |  |  |  |  |  |  |  |  |  |  |  |  |  |  |  |
|  | Balgkouranidou 2014 | NA | NA | NA | NA | NA | Greece | Caucasians | MSP | 23/48 | 0/24 |  |  |  |  |  |  |  |  |  |  | 6 |
|  |  |  |  |  |  |  |  |  |  |  |  |  |  |  |  |  |  |  |  |  |  |  |
| *BLU* |  |  |  |  |  |  |  |  |  |  |  |  |  |  |  |  |  |  |  |  |  |  |
|  | Hsu 2007 | 69 | 1-4 | 13/41 | 45/18 | 37 | Taiwan | Asians | nMSP | 19/63 | 5/36 |  |  |  |  |  |  |  |  |  |  | 8 |
|  | Liu 2010 | 61 | 1-4 | 37/22 | 59/21 | 47 | China | Asians | MSP | 39/80 | 33/80 |  |  |  |  |  |  |  |  |  |  | 8 |
|  |  |  |  |  |  |  |  |  |  |  |  |  |  |  |  |  |  |  |  |  |  |  |
| *KLK10* | |  |  |  |  |  |  |  |  |  |  |  |  |  |  |  |  |  |  |  |  |  |
|  | Zhang 2011 | NA | 1-2 | NA | NA | NA | China | Asians | MSP | 32/110 | 2/50 |  |  |  |  |  |  |  |  |  |  | 7 |
|  |  |  |  |  |  |  |  |  |  |  |  |  |  |  |  |  |  |  |  |  |  |  |
| *RASSF2* | |  |  |  |  |  |  |  |  |  |  |  |  |  |  |  |  |  |  |  |  |  |
|  | Zhao 2013 | 59.6 | 1-4 | 27/35 | 40/22 | NA | China | Asians | PCR | 24/62 | 0/46 |  |  | 11/41 | 13/21 |  |  |  |  |  |  | 7 |
|  |  |  |  |  |  |  |  |  |  |  |  |  |  |  |  |  |  |  |  |  |  |  |
| *DCLK1* | |  |  |  |  |  |  |  |  |  |  |  |  |  |  |  |  |  |  |  |  |  |
|  | Powro´zek 2015 | NA | 2-4 | 20/22 | NA | NA | Poland | Caucasians | qMSP | 18/46 | 8/95 |  |  |  |  |  |  |  |  | 8/20 | 8/22 | 7 |
|  |  |  |  |  |  |  |  |  |  |  |  |  |  |  |  |  |  |  |  |  |  |  |
| *AIM1* | |  |  |  |  |  |  |  |  |  |  |  |  |  |  |  |  |  |  |  |  |  |
|  | Begum 2011 | 65 | 1-4 | 26/36 | 40/36 | NA | USA | Caucasians | qMSP | 14/76 | 1/30 |  |  |  |  |  |  |  |  | 4/26 | 8/36 | 7 |
|  |  |  |  |  |  |  |  |  |  |  |  |  |  |  |  |  |  |  |  |  |  |  |
| *hOGG1* | |  |  |  |  |  |  |  |  |  |  |  |  |  |  |  |  |  |  |  |  |  |
|  | Liu 2010 | 61 | 1-4 | 37/22 | 59/21 | 47 | China | Asians | MSP | 18/80 | 6/80 |  |  |  |  |  |  |  |  |  |  | 8 |
|  |  |  |  |  |  |  |  |  |  |  |  |  |  |  |  |  |  |  |  |  |  |  |
| *KIF1A* | |  |  |  |  |  |  |  |  |  |  |  |  |  |  |  |  |  |  |  |  |  |
|  | Ostrow 2010 | 69 | 1-4 | 7/47 | 37/33 | 68 | USA | Caucasians | qMSP | 13/70 | 1/80 |  |  |  |  |  |  |  |  |  |  | 8 |
|  |  |  |  |  |  |  |  |  |  |  |  |  |  |  |  |  |  |  |  |  |  |  |
| *NISCH* | |  |  |  |  |  |  |  |  |  |  |  |  |  |  |  |  |  |  |  |  |  |
|  | Ostrow 2010 | 69 | 1-4 | 7/47 | 37/33 | 68 | USA | Caucasians | qMSP | 29/70 | 20/80 |  |  |  |  |  |  |  |  |  |  | 8 |
|  |  |  |  |  |  |  |  |  |  |  |  |  |  |  |  |  |  |  |  |  |  |  |
| *SEMA3B* | |  |  |  |  |  |  |  |  |  |  |  |  |  |  |  |  |  |  |  |  |  |
|  | Liu 2010 | 61 | 1-4 | 37/22 | 59/21 | 47 | China | Asians | MSP | 73/80 | 38/80 |  |  |  |  |  |  |  |  |  |  | 8 |

NA: not applicable; NSCLC: non-small cell lung cancer; AC: adenocarcinoma; SCC: squamous cell carcinoma; MSP: Methylation Specific PCR; PCR: Polymerase Chain Reaction; qMSP; quantitative Methylation Specific PCR; nMSP: nested Methylation Specific PCR; FMSP: fluorescent Methylation-specific PCR; M+: the number of gene methylation; N: the number of samples; NOS: Newcastle–Ottawa Scale.
